# Comparative metabolomics analysis of milk components between Italian Mediterranean buffaloes and Chinese Holstein cows based on LC-MS/MS technology

**DOI:** 10.1371/journal.pone.0262878

**Published:** 2022-01-25

**Authors:** Xiang Yuan, Wen Shi, Jianping Jiang, Zhipeng Li, Penghui Fu, Chunyan Yang, Saif ur Rehman, Alfredo Pauciullo, Qingyou Liu, Deshun Shi

**Affiliations:** 1 State Key Laboratory for Conservation and Utilization of Subtropical Agro-Bioresources, Guangxi University, Nanning, China; 2 Guangxi Engineering Technology Research Center of Chinese Medicinal Materials Stock Breeding, Guangxi Botanical Garden of Medicinal Plants, Nanning, China; 3 Department of Agricultural, Forest and Food Sciences, University of Torino, Grugliasco (TO), Italy; Universita degli Studi del Molise, ITALY

## Abstract

Buffalo and cow milk have a very different composition in terms of fat, protein, and total solids. For a better knowledge of such a difference, the milk metabolic profiles and characteristics of metabolites was investigated in Italian Mediterranean buffaloes and Chinese Holstein cows were investigated by liquid chromatography tandem-mass spectrometry (LC-MS/MS) in this study. Totally, 23 differential metabolites were identified to be significantly different in the milk from the two species of which 15 were up-regulated and 8 down-regulated in Italian Mediterranean buffaloes. Metabolic pathway analysis revealed that 4 metabolites (choline, acetylcholine, nicotinamide and uric acid) were significantly enriched in glycerophospholipid metabolism, nicotinate and nicotinamide metabolism, glycine, serine and threonine metabolism, as well as purine metabolism. The results provided further insights for a deep understanding of the potential metabolic mechanisms responsible for the different performance of Italian Mediterranean buffaloes’ and Chinese Holstein cows’ milk. The findings will offer new tools for the improvement and novel directions for the development of dairy industry.

## 1. Introduction

Milk and dairy products demanded as an important source of energy and nutrients for human nutrition has changed in the last decades, pointing to healthier products with higher nutritional values and immunological benefits. Among domestic ruminants, buffalo and cow differ in their milk composition in terms of fat and total solids, especially protein content (4.3% vs 3%) [1–4]. Italian Mediterranean buffalo (Bubalus bubalis) is an excellent buffalo breed with the highest milk yield (2000–2400 kg in 305 days of milking) [5–9], and relative high fat content (8%-9%) and protein content (4%-5%) (http://www.gxbri.com/index.htm). One of the most famous Italian dairy products “Mozzarella di bufala Campana” is processed from Italian Mediterranean buffalo milk, which is honored by the Protected Designation of Origin (PDO) label since 1996 [10, 11]. Meanwhile, buffalo has been traditionally considered also less susceptible than cattle to inflammatory diseases such as mastitis [12, 13].

The particular biological characteristics between the two kinds of milk may be due to the differences in metabolic mechanisms of the two species (buffalo *vs* cow), and there is desire to investigate these differences in the light of new available technologies. In this respect, along with the development of informatics in support of new analytical methods, metabolomics has revolutionized the metabolite profiling and biomarker identification [14]. Investigations have demonstrated that the metabolites, as the products of metabolism, are involved in essential biological process, such as immune disease, nutrition, etc. [14]. Moreover, metabolic profiles have provided insight into the mechanism that underlying various economic traits, including the milk production [15, 16], heat stress [17], feed efficiency [18, 19], flavor [20] and negative energy balance [21]. Earlier evidences have proved that metabolites had strong correlation with milk composition traits in dairy cattle [15, 22, 23], and several metabolites, such as choline and succinic acid could represent biomarkers to distinguish the Holstein milk from those of Jersey, buffalo, yak, and goat [24, 25]. These findings indicated that the intrinsic metabolic status could enlighten important metabolic processes and essential cellular functions.

In the present study, we adopted a metabolomics approach to investigate the milk metabolic profiles of Italian Mediterranean buffaloes and Chinese Holstein cows aiming to detect the metabolites associated with milk content, which could provide valuable information to illustrate the metabolic mechanism at the basis of the different milk performances of the two species.

## 2. Materials and methods

### 2.1. Ethics statement

All the research work was conducted were in compliance with the institutional guidelines and under a protocol approved by the Animal Experimental Ethical Inspection committee of Guangxi University (Gxu-2021-111).

### 2.2. Animals

A total of thirty individuals of Italian Mediterranean buffaloes (n = 15) and Chinese Holstein cows (n = 15) were chosen from Guangxi Huaxu Buffalo Biological Technology Co., Ltd. and the dairy farm of Guangxi University, respectively. All the selected animals were of similar parity (2 or 3) were in middle lactation (100-150d) and housed in the same living, management and feeding conditions for an adjustment period of 20 days before experimental milk collections. All animals were milked and fed twice a day (6:00–9:00 am and 14:00–15:00 pm) with the nutrient diet (S1 Table), whereas water was always available during the whole experimental period. Milk samples were collected in 50 mL sterilized tubes kept on ice bath, then transported to laboratory, divided into 2 mL aliquots and stored at -80°C for further analysis.

### 2.3. Analysis of milk composition and extraction of metabolites

Milk composition and somatic cell count (SCC) were analyzed by MilkoScanTM FT120 (Foss Electric A/S, Hillerød, Denmark). Metabolites were extracted using the method previously described [26, 27]. Briefly, milk samples were thawed at room temperature, then 50 μL from each sample was mixed with 350 μL of chilled extraction liquid, Methanol: Methyl‐tert‐butyl ether (MTBE) in the ratio 1:1 (vol: vol), containing Vitamin E acetate (25 ppm) as internal standard. The mixture was vortexed for 3 min, subsequently incubated at 4 °C for 10 min and centrifuged at 14,000 g for 15 min at 4 °C. The supernatant was transferred to a 0.22 μm filter (Corning, SLC, USA), and centrifuged at 14,000 g for 5 min at 4 °C. Finally, clear supernatant was used for analysis.

### 2.4. LC–MS/MS analysis

LC-MS/MS analysis was performed based on the Dionex UltiMate 3000 Uhplc system equipped with Q Exactive mass spectrometer (Thermo Fisher Scientific, CA, USA) operating in data-dependent acquisition (DDA) mode. Samples were injected onto a Hypersil GOLD HPLC column (50×2.1mm, 1.9μm). The mobile phase consisted of a gradient system of (A) 10mM ammonium formate in water and (B) 10mM ammonium formate in methanol: 0–2 min,5% B; 2–13 min, 5–95% B; 13–16 min, 95% B; 16.1-18min, 5% B; delivered at 0.3 mL/min.

The electrospray ionization (ESI) source parameters were set as follows: spray voltage of Q-Exactive mass spectrometer was set at 3.5kV/3.2kV in positive/negative polarity mode and capillary temperature was 320 °C. Sheath and aux gas flow rates were set as 30psi and 10 arb, respectively. Liquid chromatography-high resolution mass spectrometry (LC-HRMS) was operated with full scan + data dependent MS2 mode. The MS data was obtained with a scan range from 100–1000 m/z at a resolution of 70,000. The automatic gain control (AGC) target value was 300,000 and the maximum ion injection time was 100 ms. MS2 fragmentation was carried out with a resolution of 17,500 with AGC target value at 100,000 and dynamic exclusion for 10 s. Stepped collision energy (CE) fragmentation was achieved in the HCD cell at three values of normalized collision energy (NCE), namely, 40-60-80 NCE in positive mode and negative mode.

Raw LC-MS/MS data were analyzed by Compound Discoverer v. 3.0 (Thermo Fisher Scientific, CA, USA) with an untargeted metabolomics workflow: spectra selection, blank subtraction, peak picking and retention time (RT) alignment, candidate comparison with the mzCloud and ChemSpider database.

### 2.5. Data analysis

The milk composition data were statically analyzed by using Student’s t-test by SPSS version 22.0 (IBM Corp., Armonk, NY). Multivariate statistical analysis including principal component analysis (PCA) and orthogonal partial least-squares discriminant analysis (OPLS-DA) was executed to visualize the metabolic variations using SIMCA-P v.14.1 (Umetrics, Umeå, Sweden) after pareto (Par) scaling. The quality of OPLS-DA model was evaluated by cumulative parameters R^2^X, R^2^Y, and Q^2^ in cross-validation, and adopting a permutation test with 200 permutations.

Differential metabolites were identified using the variable influence on projection (VIP) values > 1, *P*< 0.05 and |log_2_ (fold change) |>1. Hierarchical cluster analysis (HCA) was performed by applying RStudio v.1.4.1106 (package pheatmap). Afterward, correlation network analysis and metabolic pathway enrichment were carried out according to annotation in Kyoto Encyclopedia of Genes and Genomes (KEGG) through MetaboAnalyst v.4.0 (https://www.metaboanalyst.ca/) [28]. The correlation analysis between metabolites and milk compositions was performed with single-Y Orthogonal extension of Partial Least-Squares (OPLS) regression using SIMCA-P v.14.1 (Umetrics, Umeå, Sweden).

## 3. Results

### 3.1. Milk composition of Italian Mediterranean buffaloes and Chinese Holstein cows

Descriptive statistical analysis evidenced that milk fat percentage, protein percentage and total solid of Italian Mediterranean buffaloes were higher than those of Chinese Holstein cows, otherwise with lower SCC of Italian Mediterranean buffaloes; whereas, as expected, the content of lactose did not show differences between milk of Italian Mediterranean buffaloes and Chinese Holstein cows (Table 1).

**Table 1:** Descriptive data of milk composition and SCC of buffaloes and cows.

Sample	Fat/%	Protein/%	Lactose/%	TS/%	SCC
					1×10^4^/mL
Buffaloes	8.60±0.44^a^	4.38±0.13^a^	5.10±0.07^a^	18.88±0.51^a^	6.40±1.00^a^
Cows	4.18±0.26^b^	2.95±0.10^b^	5.03±0.05^a^	12.70±0.30^b^	12.39±1.63^b^

1 ^a, b^ Mean values in the same column with different superscripts (*P* < 0.05) differ between the two groups.

2 TS: Total solid.

### 3.2. Metabolic profiles and multivariate analysis

**Fig 1:**
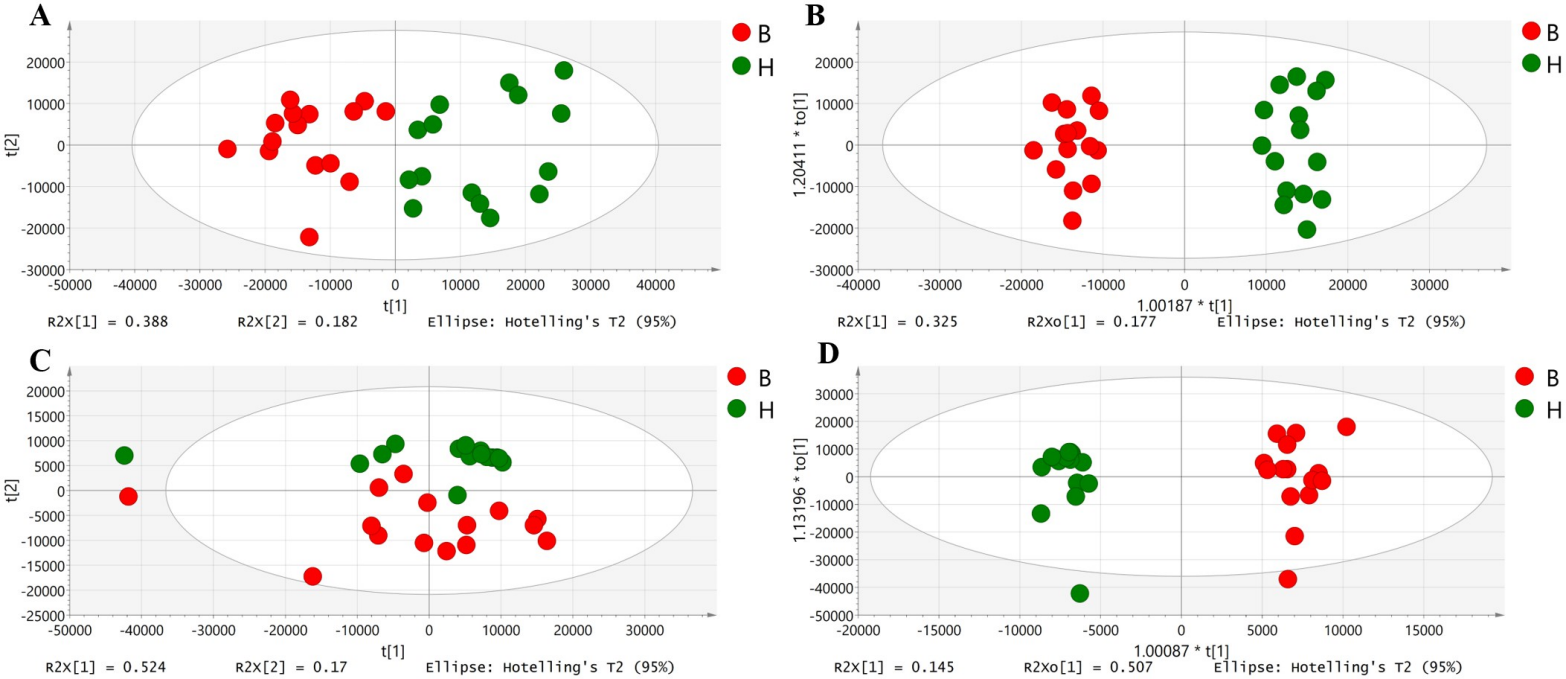
PCA (A and B, in positive and negative modes, respectively) and OPLS-DA (C and D, positive and negative modes, respectively) scores plots of Italian Mediterranean buffaloes’ milk and Chinese Holstein cows’ milk. The red circle represents buffaloes, while the green circle represents cows. PCA = principal component analysis. OPLS-DA = orthogonal partial least squares discriminant analysis.

Totally, 990 and 222 peaks were acquired in positive and negative ion modes, respectively. PCA analysis was conducted to determine the global differences between the metabolic profiles of the two groups. As depicted in Fig 1A and 1B, a clear separation between buffaloes and cows was detected. The R^2^X (cum) and Q^2^ ranged from 0.787 to 0.827 and from 0.448 to 0.576, respectively. Subsequently, OPLS-DA models demonstrated a distinct discrimination between the two groups (Fig 1C and 1D). The satisfactory values for the intercepts (in the positive ion mode, R^2^X (cum) = 0.651, R^2^Y (cum) = 0.973, Q^2^ = 0.935; in the negative ion mode, R^2^X (cum) = 0.815, R^2^Y (cum) = 0.976, Q^2^ = 0.923) indicated that OPLS-DA models were stable and valid. Then, model cross-validation through permutation tests (200 times) generated the intercepts of R^2^ and Q^2^ (positive ion mode, 0.547 and -0.597; negative ion mode, 0.588 and -0.763, respectively) (S1 Fig). The data presented herein demonstrated that a clear and significant separation existed between Italian Mediterranean buffaloes and Chinese Holstein cows by multivariate analyses (PCA and OPLS-DA).

### 3.3. Identification of differential metabolites

**Fig 2:**
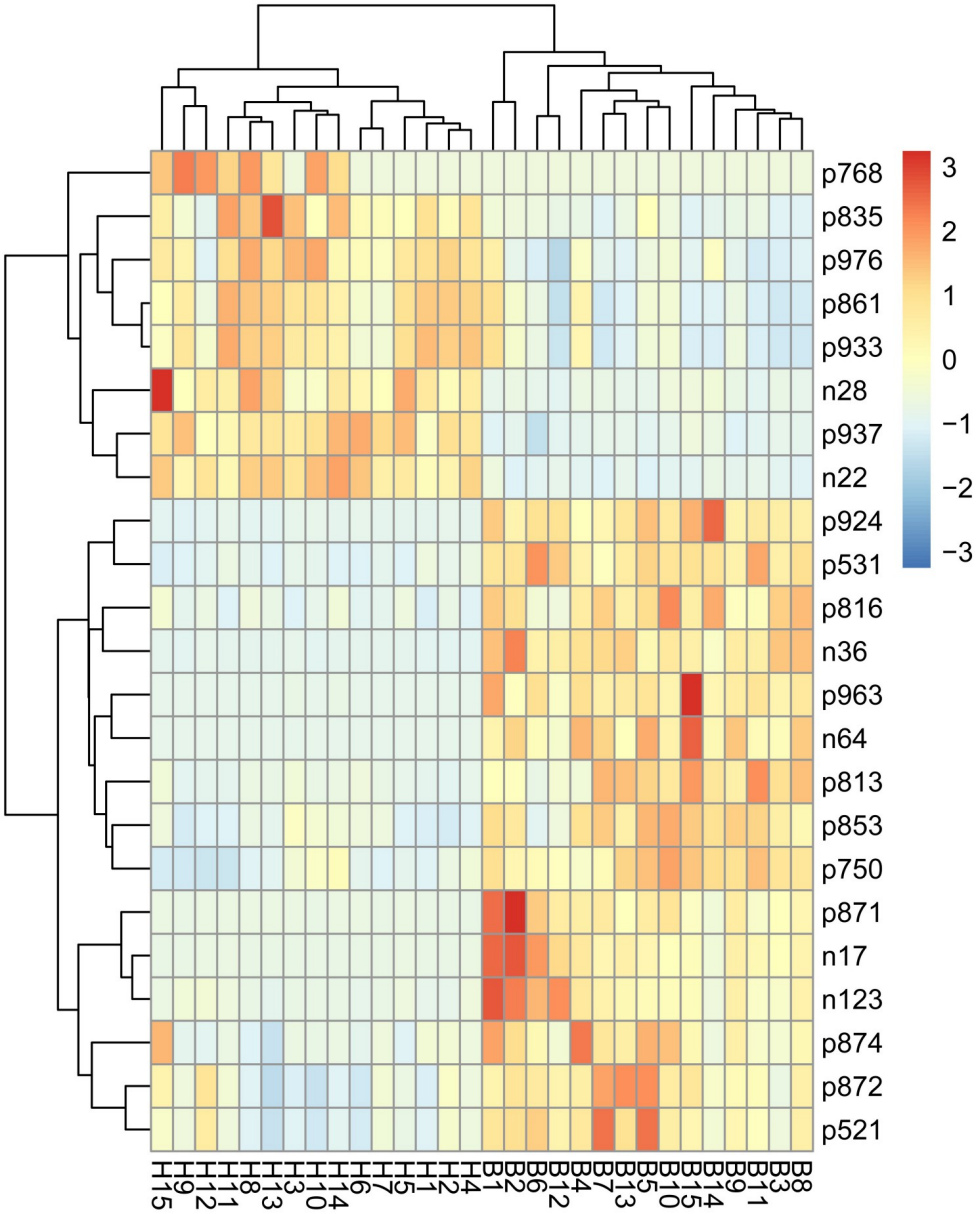
Heatmap of significantly differential metabolites between Italian Mediterranean buffaloes’ milk and Chinese Holstein cows’ milk. The horizontal axis represents milk samples; The vertical axis represents metabolites.

We identified 23 significantly differential metabolites between the two groups based on the screening criteria with VIP > 1, *P*< 0.05 and |log_2_ FC|>1. In particular, 15 metabolites were up-regulated and 8 were down-regulated in Italian Mediterranean buffalo *vs* Chinese Holstein cows (Table 2). Hierarchical clustering analysis showed that each type of the two groups exhibited a distinct metabolic pattern (Fig 2).

**Table 2:** Significantly different milk metabolites up- and down-regulated in Italian Mediterranean buffaloes *vs* Chinese Holstein cows.

	No.	Metabolites	MW(Da)	RT(min)	Log_2_FC	*P*-value	VIP
Up	p853	DL-Carnitine	161.105	0.477	2.31	2.71E-02	7.36
	p816	Acetyl-β-methylcholine	159.125	0.507	2.17	4.39E-02	6.04
	p813	Acetylcholine	145.109	0.482	2.94	3.03E-03	4.51
	p872	Glyceryl 1,2-Dicaprate	400.317	14.845	1.72	2.07E-03	4.51
	p924	N-(tert-Butoxycarbonyl)-L-leucine	231.146	2.394	3.17	1.76E-04	4.42
	p521	(2S)-2,3-Dihydroxypropyl (11Z,14Z)-11,14-icosadienoate	382.307	14.834	2.08	1.20E-04	3.46
	p531	(R)-3-hydroxybutyrylcarnitine	247.141	0.697	3.11	8.69E-04	2.55
	p963	salinosporamide B	279.146	5.272	5.57	7.25E-08	2.38
	p750	2-methylbutyrylcarnitine	245.162	4.98	2.58	3.05E-04	1.87
	p871	Glycerophospho-N-palmitoyl ethanolamine	453.284	14.014	3.75	6.78E-08	1.76
	p874	glycidyl oleate	338.281	14.966	1.30	3.38E-03	1.35
	n17	1-oleoyl-sn-glycero-3-phosphoethanolamine	479.300	14.373	3.74	4.83E-07	3.50
	n36	1-stearoyl-sn-glycero-3-phosphoethanolamine	481.316	14.294	3.82	5.58E-08	3.04
	n64	2-[(5-Amino-1, 3, 4-thiadiazol-2-yl)thio]-N-(8-methyl-8-azabicyclo[3.2.1]oct-3-yl)acetamide	313.102	2.595	6.31	6.00E-11	2.00
	n123	1-linoleoyl-sn-glycero-3-phosphoethanolamine	477.285	14.042	3.18	1.33E-05	1.09
Dw	p835	Choline	103.100	0.468	-2.03	2.57E-03	3.68
	p861	Ethanoic anhydride	102.031	0.504	-1.84	3.78E-02	2.04
	p933	N-Acetyl-α-D-glucosamine	180.062	0.504	-2.00	1.49E-02	1.90
	p976	Trigonelline	137.047	0.504	-1.32	3.98E-02	1.86
	p768	3-Hydroxy-5-methoxy-6-methyl-2,3-dihydro-4H-pyran-4-one	158.057	3.916	-6.67	7.34E-03	1.71
	p937	Nicotinamide	122.048	0.66	-1.33	3.69E-03	1.33
	n22	Uric acid	168.027	0.639	-3.37	9.58E-04	4.00
	n28	Indole-3-carboxilic acid-O-sulphate	241.004	5.38	-2.27	1.78E-02	2.47

### 3.4. Correlation network and metabolic pathway analyses

**Fig 3:**
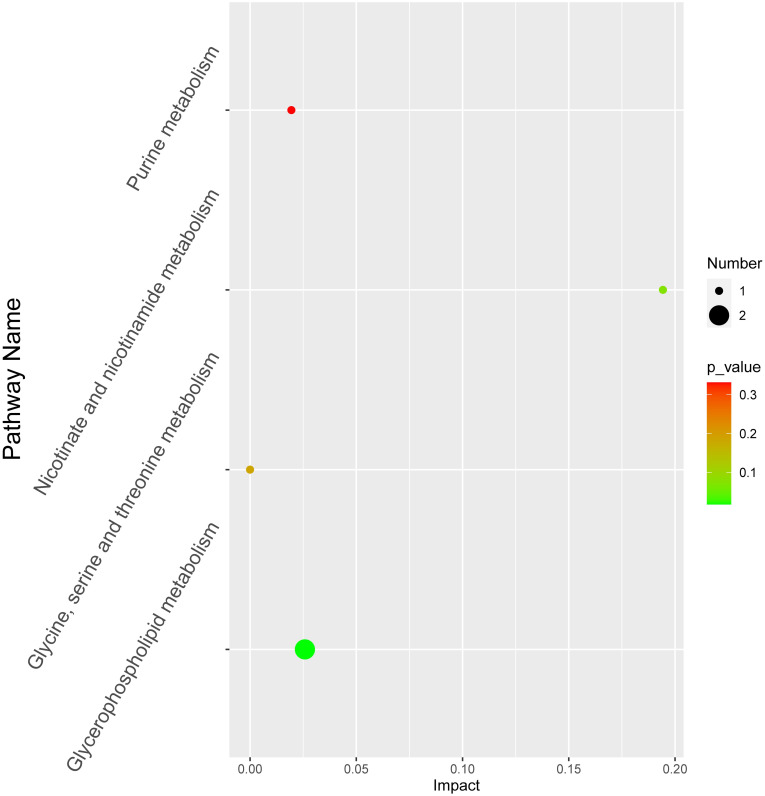
Metabolic pathways for 23 significantly differential regulated metabolites. The x-axis represents the pathway effect, and the y-axis represents the pathway name. Large sizes and dark colors represent the number of metabolites and p-value, respectively.

**Fig 4:**
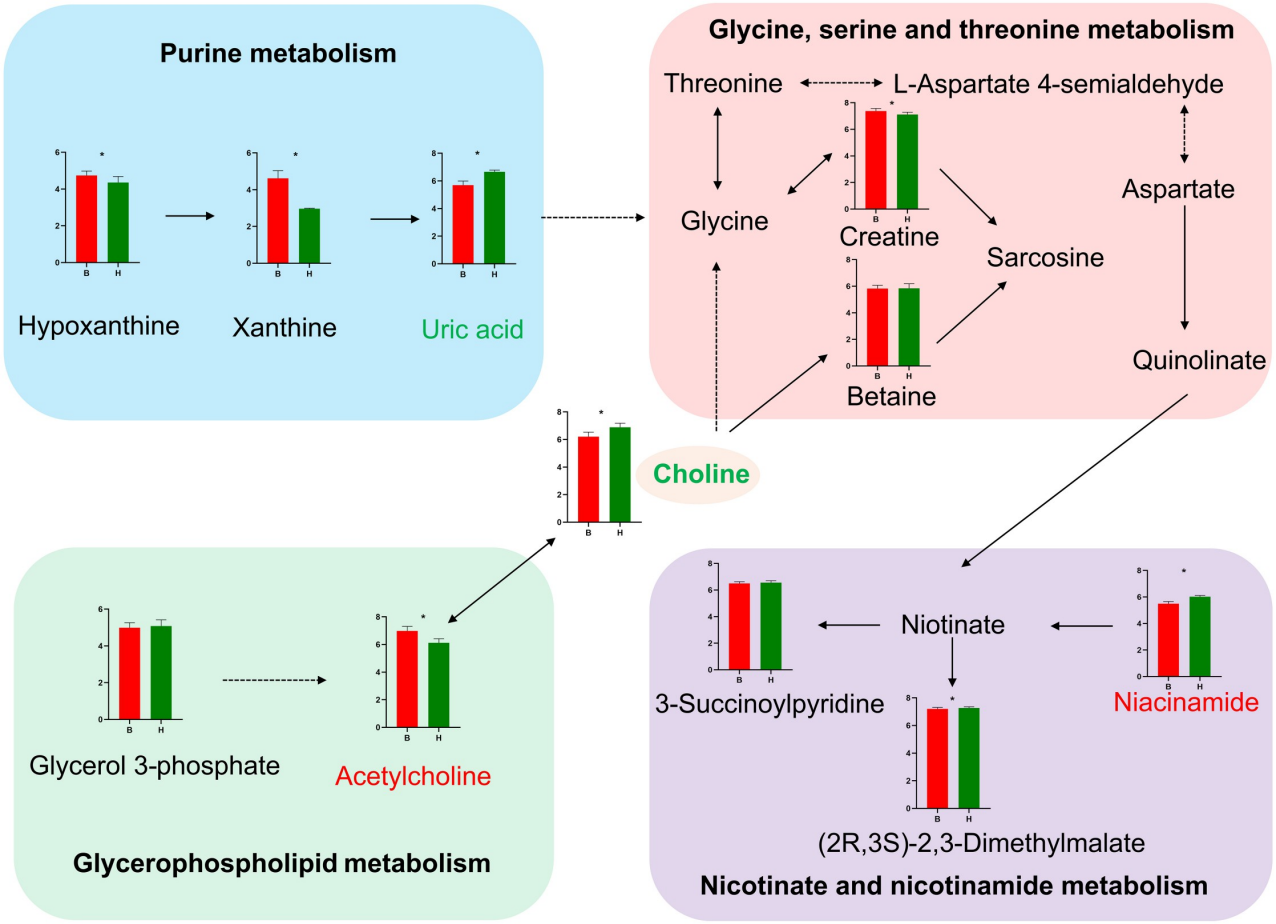
Hypothesized pathway map of the different milk performance between Italian Mediterranean buffaloes (red histograms) and Chinese Holstein cows (green histograms). Metabolites with bars were identified in current study, among them, *Asterisks represent metabolites those significantly different between two groups with p<0.05; Up-regulated and down-regulated metabolites screened by selection criteria are also indicated in red or green font, metabolites with black font were with VIP<1. Four metabolic pathways are shown in boxes. The dotted line represents the indirect regulatory relationship and the solid line represents the direct regulatory relationship. Additionally, in the histogram, red on the x-axis represents Italian Mediterranean buffaloes, whereas green represents Chinese Holstein cows. The y-axis stands for the relative concentration of metabolites between the two groups.

To investigate the interactions of the significantly different metabolites, we performed correlation network analysis. Five differential metabolites had strong correlation (S2 Fig), whereas the pathway analysis of the complete set of 23 differentially were enriched with four main biochemical pathways. Two metabolites (choline and acetylcholine) were involved in glycerophospholipid metabolism. Each metabolite was in three pathways (nicotinate and nicotinamide metabolism, glycine, serine and threonine metabolism and purine metabolism) (Fig 3). Consequently, a hypothesized pathway map based on the above four key pathways, including the corresponding differentially regulated metabolites, was integrated and proposed (Fig 4).

## 4. Discussion

In the last decades, the interest and demand for the buffalo milk has increased worldwide due to its high-quality characteristics with a higher content of fat, protein, and total solids [3, 4] and better dairy processing performances compared to bovine milk [29, 30]. Despite of these positive aspects, buffaloes produce lower milk yield. The understanding of the biological mechanisms behind these differences between the buffaloes and bovines gives rise to the interest of many researchers [31, 32]. Previously, potential protein markers and intestinal microbiome have been positively associated with differences in milk components between buffaloes and cows [33]. Inspite of the increasing advance metabolomics technologies application, still very limited information is available on the metabolic mechanism addressing the phenomenon. Therefore, in the present study, we established the milk metabolic profiles of Italian Mediterranean buffaloes and Chinese Holstein cows, and identified 23 differential metabolites between the two groups.

The analysis demonstrated that four metabolic pathways, including glycerophospholipid metabolism, nicotinate and nicotinamide metabolism, glycine, serine and threonine metabolism, as well as purine metabolism that might subsequently affect milk content. Notably, four differentially regulated metabolites (acetylcholine, choline, nicotinamide and uric acid) might be potential biomarkers for the prediction of milk content.

The acetylcholine is one of the metabolites synthesized by choline [34]. Several studies have stated that acetylcholine, acting as a neurotransmitter or an immune modulator, has beneficial effects on the reduction of the oxidative stress, inflammation and apoptosis in a variety of human diseases [35, 36]. A recent study also suggested that acetylcholine is required for the acute inflammatory response [37]. In our study, the content of this metabolite was higher in buffalo milk than cow milk which might a higher resistance of buffaloes for the mastitis [13] and confirm that acetylcholine might have an essential role in mammary immune response, a higher concentration of acetylcholine in milk might act as an anti-inflammatory agent for maintaining the health status of mammary gland.

Generally, choline is synthesized in the liver and released into blood. It has various functions in biological processes such as cellular maintenance, cell growth and development [38]. However, it was reported that choline is also synthesized in mammary gland and located in the bilayer membrane of the milk fat globule [39, 40]. Furthermore, independent studies have proved that choline is an essential compound for hepatic lipid metabolism in dairy cattle [41, 42]. Investigations on dairy performance of lactating cows showed that milk composition was affected by choline supplementation [43, 44]. Furthermore, a previous study has reported that the relative concentration of choline in Holstein milk was higher than buffalo milk, which is consistent with our observation [45]. Besides, choline as a potential metabolic biomarker has a central role in mammary immune response and it was positively correlated with mastitis [40, 46].

The nicotinamide was differentially regulated between Italian Mediterranean buffalo and Chinese Holstein was the nicotinamide. This metabolite acts as an important precursor of NAD+ in milk [47], and it can interacted with NAD+ and NADP+ to play a fundamental role in the glycolysis and the TCA cycle [48]. Cervantes et al. (1996) investigated the role of the nicotinamide on the improvement of milk traits in dairy cows. This metabolite could increase the production of milk and protein, decrease fat percentage, but had no effect on either production of Fat-corrected Milk (FCM) and percentage of protein [49]. Later on, Zak et al. (2006) observed the involvement of the nicotinamide in lipid metabolism [50], whereas more recently a strong positive correlation was detected between the nicotinamide and milk protein yield in Holstein dairy cows [51]. These findings seem to confirm that a higher content of nicotinamide might provide more energy for milk production and explain the differences found between the Chinese Holstein cows and the Italian Mediterranean buffaloes.

Uric acid, as one of the purine derivative elements, has considered a reliable indicator to comprehensively assess cows’ healthy status and mammary energy status [52–54]. Furthermore, as an antioxidant entity, uric acid contributes to increase the oxidative stability of milk [55]. In relation to milk production, previous researches indicated that uric acid was linearly associated with milk yield in dairy cows [56, 57]. Giesecke et al. already in the 1994, have detected a strongly positive correlation between energy intake and uric acid excretion in milk, thus indicating the increasingly conversion rate of mammary purine with higher milk yield of dairy cows [58]. Therefore, our findings confirmed that the uric acid might be considered as an important metabolite supporting a higher milk fat content in Chinese Holstein cows *vs* Italian Mediterranean buffaloes.

Based on single and total metabolites pathways and their differential regulation, we manually integrated an overview pathway map and linked the information together (Fig 4). Results indicated that four significant pathways including glycerophospholipid metabolism, nicotinate and nicotinamide metabolism, glycine, serine and threonine metabolism, purine metabolism, were interacted together. This was in line with the result of correlation network analysis of the identified metabolites. Therefore, we hypothesized that the alteration of the four metabolic pathways could have significant impact on this milk content trait. Definitely, further investigation on the four key-metabolites should be conducted to consider their concrete role in milk metabolism and to develop an integrated metabolic biomarker map for the understanding and improvement of milk performances of dairy animals. However, taken together, our results provided for the first-time useful information to elucidate the metabolic mechanisms of two dairy species with very different milk performances for both milk yield and composition.

## 5. Conclusions

This study investigated the milk metabolic profiles of Italian Mediterranean buffaloes and Chinese Holstein by LC-MS/MS. Four metabolites, including acetylcholine, choline, nicotinamide and uric acid are related to the differences in milk content traits between Italian Mediterranean buffaloes and Chinese Holstein. This finding will provide a new route to improve the milk content traits in dairy species.

## Supporting information

S1 Fig OPLS-DA permutation test in positive (A) and negative ion mode (B). (DOCX)

S2 Fig Correlation network of 24 significantly differential regulated metabolites between Italian Mediterranean buffaloes and Chinese Holstein cows. (DOCX)

S1 Table Feed composition and nutrient levels of diets, % (air-dry basis). (DOCX)
