# Supplementary figures and images for: Comparative metabolomics analysis of milk components between Italian Mediterranean buffaloes and Chinese Holstein cows based on LC-MS/MS technology
Source: PLoS One. 2022 Jan 25;17(1):e0262878. doi: 10.1371/journal.pone.0262878 (PMC8789157; doi:10.1371/journal.pone.0262878)

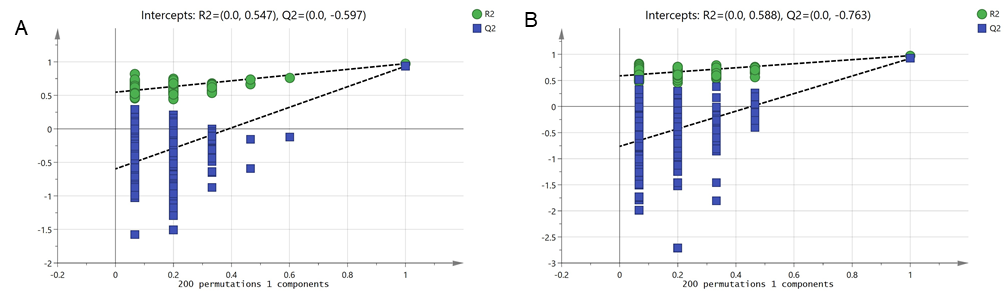


**S1 Fig.** OPLS-DA permutation test in positive (A) and negative ion mode (B).

Supplement: S1 Fig — (DOCX) [file pone.0262878.s001.docx]
