# Supplementary material for: Comparative metabolomics analysis of milk components between Italian Mediterranean buffaloes and Chinese Holstein cows based on LC-MS/MS technology
Source: PLoS One. 2022 Jan 25;17(1):e0262878. doi: 10.1371/journal.pone.0262878 (PMC8789157; doi:10.1371/journal.pone.0262878)
Supplement: S2 Fig — (DOCX) [file pone.0262878.s002.docx]

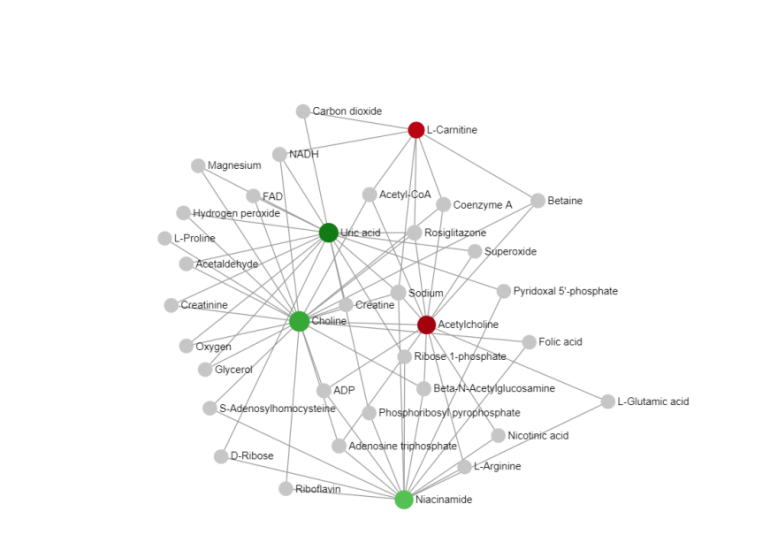


**S2 Fig.** Correlation network of 24 significantly differential regulated metabolites between Italian Mediterranean buffaloes and Chinese Holstein cows
