# Supplementary material for: Comparative metabolomics analysis of milk components between Italian Mediterranean buffaloes and Chinese Holstein cows based on LC-MS/MS technology
Source: PLoS One. 2022 Jan 25;17(1):e0262878. doi: 10.1371/journal.pone.0262878 (PMC8789157; doi:10.1371/journal.pone.0262878)
Supplement: S1 Table — (DOCX) [file pone.0262878.s003.docx]

**S1 Table.** Feed composition and nutrient levels of diets, % (air-dry basis).

| **Items** | **Content/%** |
| --- | --- |
| Elephant grass | 12 |
| Beer slack | 21 |
| Cassava residue | 33 |
| Corn | 17.83 |
| Wheat bran | 7.51 |
| Soybean meal | 5.72 |
| Lime stone | 0.5 |
| CaHPO4 | 0.6 |
| NaHCO3 | 0.8 |
| NaCl | 0.7 |
| Premix | 0.34 |
| Total | 100 |
| GE/（MJ/Kg） | 16.8 |
| CP | 14.6 |
| NDF | 36.21 |
| ADF | 23.5 |

^1.^The additive premix provided the following per Kg of diets: VA 550 000 IU, VE 3000 IU, VD3 150 000IU, 4.0g Fe (as ferrous sulfate), 1.3g Cu (as copper sulfate), 3.0g Mn (as manganese sulfate), 6.0g Zn (as zinc sulfate), 80mg Co(as cobalt sulfate).
